# Team-based learning versus Traditional lecture-based learning: An investigation of students’ perceptions and academic achievements

**DOI:** 10.12669/pjms.37.4.4000

**Published:** 2021

**Authors:** Shazia Nawabi, Rabia Bilal, Muhammad Qasim Javed

**Affiliations:** 1 Shazia Nawabi, Associate Professor, Department of Prosthodontics, College of Dentistry, Qassim University, Saudi Arabia; 2 Rabia Bilal, Associate Professor, Department of Orthodontics, College of Dentistry, Qassim University, Saudi Arabia; 3 Muhammad Qasim Javed, Assistant Professor, Department of Conservative Dental Sciences, College of Dentistry, Qassim University, Saudi Arabia

**Keywords:** Academic achievement, Assessment, Lectures, Team-based learning

## Abstract

**Objectives:**

Evaluation of TBL from students’ perspective has been done extensively, but limited studies have analyzed the effect of TBL on students’ academic performance. Objectives of the present study were to assess students’ perceptions about team-based learning versus traditional lectures and then to compare with students’ grades in both TBL and lecture-based assessments.

**Methods:**

Cross-sectional descriptive study which included 147 dentistry students was conducted between March and June, 2020 at Qassim University, Saudi Arabia. A self-composed 10 item closed ended instrument was administered through electronic mail. One block from each academic year was selected randomly and organizers were requested to provide grades of students in both TBL and lecture-based exams. Grades from A to F were determined as achievement indicator and were compared statistically using Kruskal Wallis, Tuckey Posthoc and Chi-square tests.

**Results:**

A total of 120 students (81.6%) responded to the survey, and mean perception score was (3.29±0.94). Perceptions of students, regarding TBL as a learning strategy were comparatively superior as compared to the lectures. Students scored significantly higher grades in TBL exams (p<0.05) as compared to lecture-based assessments. Gender-wise analysis indicated that female students secured significantly higher grades in the TBL.

**Conclusion:**

We conclude that dentistry students perceived TBL as superior teaching and learning strategy compared to traditional lectures. Their perception was verified by their significant higher academic achievements in the assessments for the coursework taught by utilizing team-based learning strategy.

## INTRODUCTION

Fundamental goal of health-professions education is to provide a culture with an educated, skilled, and well-informed cadre of professionals who set patient care above everything and strive to maintain this attitude for lifetime. Medical education continues to make its own significant advances to produce such professionals.^1^

Lecture is commonly used method of teaching. Lectures are used to convey history, theories, critical information, and are criticized for being one-way mode of communication that does not encompass involvement of the audience leading to passive acquiring. However, medical universities have not found a viable substitute to replace lectures in curriculums as lectures are a quick, inexpensive, and efficient way of introducing complex educational material.^2^,^3^ Also, brilliant speakers can give highly stimulating lectures which can be an active mode of learning. However, lecturing is not an efficient way of promoting critical thought process, altering mind-sets, or teaching behavioural skills.^3^

This brings us to the problem at hand. While the magnitude of medical science is increasing steadfastly, faculty-student contact hours cannot be expanded in parallel. Teachers are trying to prepare students to adopt self-directed learning strategies outside the class.^1^ The acclimatization of active learning requires reduction in previously allocated lecture hours and allocating the time for active learning strategies that engage learners in thoughtful dialogue and emphasize on application rather than acquisition of knowledge.^4^

Team based learning (TBL) is a collaborative active learning approach. It consists of small groups of students who work together in class to apply content to simple or complicated problems with the feedback of the subject specialist.^5^ TBL is comprised of segments that can be taught in three step cycles. Pre-class activity, in-class activity which includes individual readiness assurance test (IRAT), group readiness assurance test (GRAT) and application exercise which is practice of concepts by engaging the students in the kind of collaboration that is expected in today’s clinical practice.^6^ This not only helps the students apply the instructor directed content to the real-world problems but also fosters content understanding.^7^ Considering this, TBL is utilized in several educational institutes and recent literature recommends TBL as a resource effective dynamic learning tool for development of critical reasoning, higher cognition, effective communication, collaborative teamwork and lifelong problem-solving skills.^7^,^8^

Evaluation of TBL from students’ perspective has been pondered but there is not much literature on the impact of TBL on actual performance indicators. The aim of the present study was to assess students’ perceptions about team-based learning versus traditional lectures and then to compare them with students’ respective grades in TBL and lecture-based assessments.

## METHODS

This cross-sectional study was conducted at Qassim University, Dental College, Saudi Arabia between March and June 2020 after the provision of ethical approval by Institutional research committee (ST/6073/2020. dated March. 23, 2020). The intended sample was 160 students of first to third academic years by utilizing nonprobability convenience sampling technique.

The Dental College is implementing hybrid active-learning curriculum at the level of multi-disciplinary integration. It is divided into blocks and each block consists of set of themes. Lectures and problem-based learning are the main medium of instruction whereas the TBL is a relatively novel approach. TBL sessions are conducted in small groups of six to eight students in the presence of a tutor and a content expert. TBL sessions have five essential components: pre-class reference-oriented work, 20 minutes in-class IRAT, followed by same test in groups (GRAT) (20 minutes), explanation session (10 minutes) and 60 minutes application-focused exercise. Reference of TBL content is sent to the students one week in advance. Results are displayed promptly after completion of both tests and immediate feedback is given by the content expert. Results of all TBL based tests in the block are compiled and added towards the end block results.

An electronic 10 item closed ended instrument was developed to assess perceptions of students by principal author on a 5-point Likert-type scale (strongly disagree to strongly agree), while face validity was established by a team of senior educationists (two internal and one external) and a psychometrician. Next the questionnaire was pilot tested on 13 students (later excluded from the study). The instrument was administered through emails to registered students.

Subsequently, three blocks were selected randomly, one from each academic year. Block organizers were requested to provide grades of students in final block assessments anonymously to safeguard privacy of students. End block exam data consisted of separate grades for both lecture and TBL based assessments. Both assessments have same format of multiple-choice questions (K1 and K2 type) based on the objectives of the blocks. Regarding reliability of assessment procedures, the MCQs are constructed by independent subject specialists, verified by relevant block organizers, and validated by evaluation and assessment unit (EAU) through calculation of internal reliability coefficient. Finally, the post-test item analysis is run to identify any item flaws.

Total number of grades from A* to F were taken as achievement indicators in both categories. SPSS-23 was used for data analysis. Descriptive statistics were recorded as percentages and frequencies. Kruskal Wallis test and Tuckey Posthoc test were used for item-based year wise analysis of mean perception scores for significant differences. Chi-square test was utilized to check the significant differences for the:

1. Item based gender-wise analysis of mean perception scores.
2. Comparison of TBL Exam grades and Lecture exam grades for individual years.
3. Gender-wise analysis for the TBL and lecture exam grades for individual years.

## RESULTS

Out of 147 students, 120 (81.6%) responded to the survey questionnaire. The lowest response rate (58%) was noted for the second-year students**.** On the other hand, the response rate for first and third-year students was 100%. The female to male ratio of the respondents was 1:1.18. The perception questionnaire was found to be reliable with Cronbach-Alpha value of 0.91.

The highest mean perception score (3.67 ± 1.19) was noted for the item 7. Whereas the lowest mean perception score (3.10±1.48) was noted for the item 8. The overall mean perception score was 3.29±0.94. The gender-based item-wise analysis showed that perception of females was significantly different from the males for the item numbers 5 and 10 (p-value<0.05). The item-based year wise analysis highlighted the significant difference in the mean perception score for the Items 5, 8 and 9 (p-value<0.05) (Table-I). Subsequently, Tuckey’s posthoc test suggested that the mean perception scores of first year students were significantly higher than the third-year students for above mentioned three items. The overall combined mean perception of first academic year was 3.55±0.93 being the highest while it was 3.32±0.90 for the second year and lowest was for third academic year 3.07±0.95. Nevertheless, no significant difference was noted in the mean overall perception between the three academic years.

**Table-I:** Students’ perception about Lectures and Team based learning.

Items	Participant’s Responses^*^ (%)					Mean Perception Score ± SD	p-value	

	SA	A	N	D	SD		Gender^1^	Academic year^2^
1 I find myself more focused during TBL activities than lectures	19.2	20	30.8	16.7	13.3	3.15± 1.28	0.10	0.18
2 I learn better in team setting	15	35.8	24.2	15	10	3.31±1.19	0.10	0.35
3 During lecture I often find myself thinking of non-related things	16.7	33.3	25	16.7	8.3	3.33± 1.18	0.77	0.65
4 I am easily distracted during traditional lectures	13.3	33.3	27.5	19.2	6.7	3.27 ± 1.12	0.37	0.41
5 I am more likely to fall asleep during lecture then TBL activities	14.2	30.8	27.5	12.5	15	3.17± 1.25	0.01*	0.02*
6 I easily remember what I learn when working in team than in lectures	21.7	37.5	25	10	5.8	3.60 ± 1.11	0.07	0.78
7 Team based learning activities are fun and I enjoy them	28.3	34.2	20	10.8	6.7	3.67 ± 1.19	0.55	0.75
8 I do better in exams in topics learned through TBL than lectures	29.2	9.2	21.7	22.5	17.5	3.10±1.48	0.60	0.02*
9 TBL help me improve my grade	29.2	7.5	32.5	20	10.8	3.24±1.35	0.001*	0.003*
10 I think TBL is better approach to learning as compared to lectures	31.7	6.7	25	18.3	18.3	3.15±1.49	0.02*	0.25

1 Chi-square test,

2 Kruskal Wallis test,

* SA=strongly agree, A=Agree, N=Neutral, D=Disagree, SD=strongly disagree.

**Note:** Students’ perceptions was assessed by giving 5 to SA, 4 to A, 3 to N, 2 to D, 1 to SD.

Comparison of academic year-wise grades between two teaching modalities showed significant difference for second and third year separately as well as overall for all the three academic years where the students scored significantly higher grades in TBL exams (p<0.05) (Table-II). Furthermore, female students secured significantly higher grades in the TBL exams for second and third year (p<0.05) and also scored higher overall grades compared to males for all three academic years (p=0.01) (Table-III).

**Table-II:** Academic year-wise comparison of grades between TBL and Lectures.

Academic Year	Instruction Method	Exam Grades (N)										P-value	P-value Lecture vs TBL

		A+	A	B+	B	C+	C	D+	D	F	Total		
First	Lecture	0	3	2	8	5	7	3	3	3	34	0.008	0.000*
	TBL	1	7	11	11	3	0	0	1	0	34		
Second	Lecture	4	4	5	14	13	10	0	6	4	60	0.001*	
	TBL	6	33	15	6	0	0	0	0	0	60		
Third	Lecture	4	8	10	10	3	5	0	1	3	44	0.001*	
	TBL	0	13	23	7	1	0	0	0	0	44		

**Table-III:** Gender comparison of the grades between TBL and lectures.

Academic Year	Instruction Method	Gender	Exam Grades(N)										P-value	P-value male vs female

			A+	A	B+	B	C+	C	D+	D	F	Total		
First	Lecture	Male	0	0	0	4	3	5	2	3	2	19	0.19	0.01*
		Female	0	3	2	4	2	2	1	0	1	15		
	TBL	Male	1	6	5	5	1	0	0	1	0	19	0.33	
		Female	0	1	6	6	2	0	0	0	0	15		
Second	Lecture	Male	1	0	3	8	8	7	0	4	1	32	0.23	
		Female	3	4	2	6	5	3	0	2	3	28		
	TBL	Male	1	13	13	5	0	0	0	0	0	32	0.002*	
		Female	5	20	2	1	0	0	0	0	0	28		
Third	Lecture	Male	1	1	5	5	1	4	0	1	3	21	0.12	
		Female	3	7	5	5	2	1	0	0	0	23		
	TBL	Male	0	0	13	7	1	0	0	0	0	21	0.01*	
		Female	0	13	10	0	0	0	0	0	0	23		

## DISCUSSION

We found overall mean perception score of 3.29±0.94. Building on this finding, our analysis suggests that students perceived team-based learning as superior learning strategy as compared to traditional lectures. This finding which is consistent with the extensive literature validates the concordance between adult learning principles and characteristics of team-based learning. Numerous studies have shown TBL as a highly structured collaborative format which augments student learning when compared to the low structured traditional lecture format.^9^,^10^ Likewise, a number of other studies have shown Students’ perception in favor of team-based learning as compared to more traditional methods like lectures.^11^-^13^

Highest mean score was noted for item stating TBL activities as enjoyable and engaging. This finding is also supported by several previous studies reporting team-based learning as enjoyable educational experience while achieving higher-level learning outcomes concomitantly.^14^-^16^ Moreover studies have verified that components of TBL are purely based on principles of constructivist educational theory making it a gifted method to strengthen healthcare education.^17^

Students further exhibited a significant positive response to item stating, “I am more likely to fall asleep during lecture then TBL activities.^”^ It is a well-established fact that traditional lectures are boring due to limited attention span^18^ and studies have shown significant association between lecture boredom and grade point average.^19^ The other consequences of lecture tedium are missing future lectures, daydreaming and use of social media during lectures.^20^ It is therefore advocated by medical education literature to make lectures interactive by designing lecture breaks including problem sets, 1-minute papers, brain storming session, open discussions, and even formative assessment to make lectures compatible with hybrid active learning curriculum.^21^,^22^

Moreover, gender-wise item-based analysis showed that perception of female students was significantly higher than males (p-value<0.05) for the items affirming TBL as better learning approach and helped improving their grades. Accordingly, the gender wise analysis of the exam grades suggested that female students secured significantly higher grades in the TBL exams (p<0.05) and also scored higher overall grades compared to males for all three academic years (p=0.01). Literature supports positive educational outcomes with team-based learning, whilst female TBL students outperforming the male students.^23^

Generally, our findings indicate that students of all three years performed significantly better in course content learned through TBL and the proportion of top grades (A*/A) was significantly higher in TBL based rather lecture based assessments. These findings are consistent with studies done by Zgheib NK et al. and Chen M et al. to determine the long-term impact of TBL on medical students’ academic performance, concluding that students achieved higher grades in course content covered through team-based learning.^24^,^25^

### Strengths of the study

outcomes of our research confirm past studies that team-based learning is an effective instructional strategy. Also, the study offers additional insight into the impact on academic achievements.

### Limitations of the study

Certainly, our study holds certain limitations. First, the study being cross-sectional could not establish the long-term impact of TBL on learning attitudes of students and consistent academic achievements. Secondly, though the internal consistency of survey was acceptable, additional validation is needed for generalizability as this survey was conducted at one institution.

## CONCLUSIONS

Our results demonstrate that addition of team-based learning in our curriculum is a welcomed instructional strategy and our experience of replacing a percentage of didactic lectures with team-based learning proved to be promising, resulting in positive perceptions about TBL as preferred teaching and learning strategy as well as superior performance of students.

### Recommendations

Further longitudinal studies with stronger methodologies are required to establish the usefulness of TBL in higher cognitive level learning as indicated by long-term academic achievement indicators.

### Authors’ Contribution:

**SN:** Design, Data Collection, Write up, Final Approval and accountable for the accuracy of the work.

**RB:** Data collection, write up.

**MQJ:** Statistical analysis, Write up, Manuscript editing

**SN** is responsible for the accuracy/integrity of the work.
